# Fathers’ Complementary Feeding Support Strengthens the Association Between Mothers’ Decision-Making Autonomy and Optimal Complementary Feeding in Nigeria

**DOI:** 10.1093/cdn/nzac098

**Published:** 2022-06-02

**Authors:** Diana Allotey, Valerie L Flax, Abiodun F Ipadeola, Sarah Kwasu, Linda S Adair, Carmina G Valle, Sujata Bose, Stephanie L Martin

**Affiliations:** Department of Nutrition, Gillings School of Global Public Health, University of North Carolina at Chapel Hill, Chapel Hill, NC, USA; RTI International, Research Triangle Park, Durham, NC, USA; Datametrics Associates Limited, Abuja, Nigeria; Alive & Thrive, Kaduna State, Kauna, Nigeria; Department of Nutrition, Gillings School of Global Public Health, University of North Carolina at Chapel Hill, Chapel Hill, NC, USA; Carolina Population Center, University of North Carolina at Chapel Hill, Chapel Hill, NC, USA; Department of Nutrition, Gillings School of Global Public Health, University of North Carolina at Chapel Hill, Chapel Hill, NC, USA; Alive & Thrive, Washington, DC, USA; Department of Nutrition, Gillings School of Global Public Health, University of North Carolina at Chapel Hill, Chapel Hill, NC, USA; Carolina Population Center, University of North Carolina at Chapel Hill, Chapel Hill, NC, USA

**Keywords:** complementary feeding, decision-making, social support, fathers, mothers, Nigeria

## Abstract

**Background:**

Evidence about the effects of mothers’ decision-making autonomy on complementary feeding is not consistent, generating hypotheses about whether complementary feeding social support moderates the relation between mothers’ decision-making autonomy and the practice of complementary feeding.

**Objectives:**

This study examined the moderation effect of fathers’ complementary feeding support on the association of mothers’ decision-making autonomy with the WHO complementary feeding indicators of minimum dietary diversity, minimum meal frequency, and minimum acceptable diet, and post hoc secondary outcomes of feeding eggs or fish the previous day. The study also examined the concordance between mothers’ and fathers’ perspectives of mothers’ autonomy and fathers’ complementary feeding support.

**Methods:**

Data were from cross-sectional surveys of 495 cohabiting parents of children aged 6–23 mo enrolled in an Alive & Thrive initiative implementation research study in Kaduna State, Nigeria. Logistic regression models were used to examine moderation, and κ statistics and 95% CIs were used to assess the concordance in reported perspectives of the parents.

**Results:**

The moderation results show that the simple slopes for decision-making were significant for minimum meal frequency, minimum acceptable diet, and feeding children fish the previous day when fathers offered ≥2 complementary feeding support actions. There were no significant findings in the moderation models for minimum dietary diversity or feeding children eggs the previous day. The findings from the concordance tests show moderate to substantial agreement (ranging from 57.6% to 76.0%) between parents’ perspectives of mothers’ autonomy, and moderate to excellent agreement (ranging from 52.1% to 89.1%) between parents’ perspectives of fathers’ complementary feeding support.

**Conclusions:**

In Nigeria, high levels of fathers’ complementary feeding support strengthen the association of mothers’ decision-making autonomy with minimum meal frequency, minimum acceptable diet, and feeding children fish the previous day.

This study was registered with clinicaltrials.gov (NCT04835662).

## Teaser

Complementary feeding support from fathers strengthens the association of mothers’ decision-making autonomy with complementary feeding practices.

## Introduction

Optimal complementary feeding practices in children aged 6–23 mo are associated with reductions in child undernutrition and mortality, and improvements in child development and economic status in adulthood (1). Optimal complementary feeding includes diverse and nutrient-dense diets, including fruits and vegetables and animal-source foods; avoiding foods of low nutrient value or with added sugar; and continued breastfeeding (2). Despite the benefits, complementary feeding is suboptimally practiced globally, with a prevalence of 29%, 52%, and 18% respectively for minimum dietary diversity, minimum meal frequency, and minimum acceptable diet indicators in 2020 (3).

Mothers’ ability to practice optimal complementary feeding is influenced by maternal autonomy in household decision-making and social support (4–8), both of which affect mothers’ access to resources and time availability to practice optimal complementary feeding (9–11). Results from several studies in low- and middle-income countries found a positive effect of maternal decision-making autonomy on complementary feeding practices (4–7, 12, 13), although 1 study found that maternal decision-making autonomy has limited impact on complementary feeding (14).

Other studies also showed that optimal complementary feeding can be hampered when there is low complementary feeding social support (4, 8, 15, 16), specifically support from fathers (16). Fathers are influential because they can encourage or deter mothers from practicing recommended complementary feeding behaviors (17), through their roles as household decision makers and as providers of resources in many contexts. Successful complementary feeding interventions often seek to strengthen fathers’ support for complementary feeding (9, 17, 18).

Although there are relations between maternal decision-making autonomy, fathers’ support, and complementary feeding, there is little evidence on how fathers’ support moderates the relation between maternal decision-making autonomy and the practice of complementary feeding. The inconsistent relation between maternal decision-making autonomy and complementary feeding practices generates hypotheses about whether complementary feeding support could moderate the relation between maternal decision-making autonomy and the practice of complementary feeding. Understanding these relations could be helpful in explaining the inconsistent findings in the maternal decision-making autonomy and complementary feeding peer-reviewed literature and could also help inform future interventions.

Furthermore, apart from some qualitative examples (19, 20), few quantitative studies have measured mothers’ autonomy in household decision-making (21, 22) or fathers’ support for complementary feeding (8, 16, 23) using perspectives from both mothers and fathers. Much of the evidence for mothers’ decision-making autonomy and fathers’ complementary feeding support is based on mothers’ reported perspectives despite previous evidence showing that excluding other influential family members such as fathers in data collection limits understanding of intervention impact (24).

To address these gaps, the objectives of the current study were to examine if the association of mothers’ decision-making autonomy with the complementary feeding practice indicators is moderated by fathers’ complementary feeding support within the context of an intervention focused on improving fathers’ involvement in complementary feeding. We hypothesized that the relation between mothers’ decision-making autonomy and the complementary feeding practice indicators is stronger in mothers who report high levels of complementary feeding support from fathers. We also examined the concordance between mothers’ and fathers’ perspectives of mothers’ autonomy in household decision-making and fathers’ complementary feeding support.

## Methods

### Description of the intervention

In collaboration with the Alive & Thrive initiative in Nigeria, I Care Women and Youth Initiative (ICARE), a local nongovernmental organization, designed and implemented a 12-mo intervention from August 2019 to July 2020 in the Igabi local government area (LGA) of Kaduna State, Nigeria. The goal was to promote optimal complementary feeding practices by improving fathers’ involvement.

As part of the intervention, community-based organizations (CBOs), religious and traditional leaders, and community health extension workers (CHEWs) were trained by ICARE and provided with resources to carry out complementary feeding social and behavior change communication (SBCC) activities. The resources included complementary feeding counseling cards for CBOs and talking points and sermon guides for religious and traditional leaders to educate fathers during meetings and religious services. CHEWs were provided with counseling cards to use during home visits to counsel and reinforce complementary feeding messages with mothers and fathers (when present). During home visits, CHEWs counseled mothers on feeding key food combinations designed to increase children's consumption of vitamin A–rich vegetables and animal source foods and provided each mother with a feeding bowl that showed nutritious foods and the quantity of food to feed infants and children at different ages. Fathers with mobile phones received weekly SMS/text messages and voice messages on complementary feeding. In addition, complementary feeding SBCC messages were broadcast through radio and TV advertisements, and mothers and fathers received leaflets with the same messages. The radio and TV ads were aired 7–8 times per day. Findings from the evaluation of the intervention, which have been documented elsewhere (25), show low reported exposure to the intervention components, with fathers’ reported exposure ranging from 11% to 26% and mothers’ reported exposure ranging from 12% to 21%. Mothers who reported exposure to intervention messages through CBOs, religious services, home visits, and on TV had increased odds of feeding their children eggs and fish the previous day. Mothers who reported exposure to intervention messages during home visits also had increased odds of feeding children diets that met the minimum meal frequency requirement. Fathers’ reported exposure was not significantly associated with any of the complementary feeding indicators.

### Study design, eligibility criteria, and sampling methods

This study used endline survey data from 495 mother and father pairs who were part of the evaluation of the Alive & Thrive intervention in Igabi LGA. The cross-sectional survey used a multistage sampling methodology and was administered to households during August to September 2020, at the end of the 12-mo intervention. The sampling methodology included a purposive selection of 6 wards in Igabi LGA. Wards are the lowest of the 3 levels of administrative boundaries in Nigeria (26). The wards were selected to reflect the ethnic, religious, and social diversity of Kaduna State. The region is a mix of diverse religions and ethnicities, with Islam and Christianity being the 2 dominant religions, whereas Hausas and Fulanis are the predominant ethnic groups (27–29). Of the 6 wards selected, 2 were urban and 4 were rural. A total of 99 communities were selected across the wards based on population proportional to size. Within the selected communities, households were sampled using the random route walk methodology.

Mothers and fathers were eligible if they had a biological child aged 6 to 23 mo, were both aged ≥18 y, and were cohabiting regardless of their marital status. Mothers who were 15–17 y of age, married, and cohabiting with their husbands were also eligible because Nigerian law considers such mothers as consenting adults. Participation in the intervention was not an inclusion criterion for fathers or mothers for the survey. The study enrolled mothers and fathers in pairs. Surveys were administered separately to mothers and fathers by trained research assistants from Datametrics Associates. The research assistants were specifically trained to ensure that the process of data collection was private, including ensuring that mothers and fathers were not present at each other's interviews. In households where there was >1 mother with a child aged 6–23 mo (i.e., polygynous households), 1 of the mothers was randomly selected for data collection.

### Ethical approval, informed consent, and data management

Ethical approval was obtained from the Kaduna State Ministry of Health Research Ethics Committee and the RTI International Institutional Review Board. The study was also registered with clinicaltrials.gov (NCT04835662).

Participants provided consent either in their homes or at a separate location of their choice, and in either Hausa or English. The trained research assistants confirmed the preferred language for consent with potential participants. The research assistants read aloud the consent forms in the language chosen by the participants, checked for participants’ understanding, and addressed questions and concerns participants had prior to seeking their consent. Written informed consent was then obtained from all participants, either by signature or thumbprint.

The survey data were collected electronically on password-protected tablets using Open Data Kit (ODK) and uploaded daily to a secure server. ODK generated unique study ID numbers for all the participants at the time of data collection.

### Measures and variables

The survey questionnaires for mothers and fathers included questions on intervention exposure, household decision-making, and social support. The mothers’ survey questionnaire also included 14 questions on infant and young child feeding (IYCF) practices adapted from the WHO IYCF questionnaire (30) (see **Supplemental Materials**), and mothers responded based on their recall of foods fed to their children on the previous day. The survey questionnaires were translated into Hausa, pretested, and finalized in 2019 in Abuja, Nigeria. Both English and Hausa versions were used during data collection, and respondents were free to choose which language was most comfortable for them. The survey questionnaires were completed within 1–1.25 h, and mother-father pairs received a cash incentive of 1200 Naira (equivalent to US$3.50 in 2020) for participation.

### Dependent variables

The primary outcome variables were based on the 2008 WHO IYCF indicators for complementary feeding (30). These indicators included minimum dietary diversity (received ≥4 of the 7 WHO food groups), minimum meal frequency (received ≥2 feedings for children aged 6–8 mo, ≥3 feedings for breastfed children aged 9–23 mo, and ≥4 feedings for nonbreastfed children of solid, semisolid, or soft foods), and minimum acceptable diet (composite indicator made up of minimum dietary diversity and minimum meal frequency) (30). The 2008 WHO IYCF guidelines for calculating minimum dietary diversity were used for this study because the 2021 WHO IYCF recommendations were not available when the study was designed and conducted. In addition to these indicators, consumption of 2 specific food groups on the previous day (feeding eggs and fish to children aged 6–23 mo), which had been promoted by the intervention (25), yielded post hoc outcome variables.

### Independent variable 1: mothers’ decision-making autonomy

#### Assessment

Mothers and fathers were asked 7 questions on usual household decision-making. The questions focused on: *1*) food purchases; *2*) child feeding; *3*) household income and expenses; *4*) large household investments; *5*) mother's ability to work outside the home; *6*) use of fathers’ cash earnings; and *7*) use of mothers’ cash earnings (see Supplemental Materials). The questions were adapted from surveys developed and administered to women and men who participated in the Bandebereho couples’ study, a gender-transformative intervention in Rwanda (22). Mothers and fathers responded to each of the 7 questions with 1 of the following response options: 1 (father makes the decision), 2 (mother makes the decision), 3 (both make the decision), or 4 (someone else makes the decision).

#### Preparation

Consistent with other studies on mothers’ decision-making autonomy from culturally patriarchal contexts (31–34), mothers’ decision-making autonomy in the Kaduna context was measured as mothers’ participation/involvement in decisions in the household and included sole decision-making as well as joint decision-making with fathers. As a result, the response options were collapsed into 2 categories comprising 1 (autonomous household decision-making: mothers make the decision or mothers and fathers jointly make the decision) and 0 (no autonomous household decision-making: father makes the decision or someone else makes the decision) for each of the 7 questions. This was followed with exploratory factor analysis (EFA) for item selection and to identify the number of factors and the underlying factor structure for the scale. In preparation for the EFA, suitability of the items in the scale for factor analyses was assessed using the Kaiser–Meyer–Olkin (KMO) test for sampling adequacy. KMO >0.5 was used as the threshold of common variance (35). The KMOs showed the data to be suitable for factor analysis. The EFA was then completed with multiple factor solution options employed: *1*) retaining all factors with eigenvalues >1, and *2*) using the scree test “elbow method” to ensure that each factor accounts for a considerable share of the total variance of the items (36, 37). A factor loading >0.40 was used as the cutoff point for the assessment; but none of the items had factor loadings <0.40, hence no items were deleted from the scales (38, 39). Cronbach α was then used to examine the internal consistency of the scales. This was interpreted as 0.70 to 0.95 indicating acceptable internal consistency (40, 41). The EFA revealed differences using mothers’ and fathers’ measurements (**Supplemental Table 1**). Mothers’ measurements indicated a bidimensional scale (2 factors with eigenvalues >1) (35, 42) composed of: “Food and feeding-related” (3 items, Cronbach α = 0.81) and “Household finance-related” (4 items, Cronbach α = 0.69) whereas fathers’ measurements indicated a unidimensional scale (1 factor with eigenvalue >1) composed of all 7 items with Cronbach α = 0.75 (Supplemental Table 1). Both measurements showed acceptable internal consistency (40, 41). In line with previous literature demonstrating that the construct of maternal autonomy is not unidimensional (33, 43), the mothers’ bidimensional scale was used in logistic regression analyses. Thus, after completion of the EFA, the key concepts in mothers’ autonomous household decision-making were represented as summary scores for the composite scale and the subscales “Food and feeding-related” and “Household finance-related.”

### Independent variable 2: fathers’ complementary feeding support

#### Assessment

This was defined by fathers’ usual involvement in a set of 7 actions to support complementary feeding: *1*) provided money for food; *2*) purchased food specifically for the child; *3*) gave advice/reminded mother/female relatives about how to feed child; *4*) fed the child himself; *5*) taught the child how to feed him/herself; *6*) washed the child's hands before child ate; and *7*) helped with other chores so that mother could prepare food/feed the child. Mothers and fathers responded yes (1) or no (0) for each of these actions. The index of actions that define fathers’ complementary feeding support was developed based on social support theory (44) and knowledge from the complementary feeding support evidence base (8, 9).

#### Preparation

Fathers’ complementary feeding support was quantified as the sum of support reported by mothers and the sum of support reported by fathers. Scores ranged from 0 to 7, with separate scores for mothers and fathers.

### Covariates

Covariates included in our analyses were chosen based on current evidence in the peer-reviewed literature on the potential influencers of complementary feeding practices in the Nigerian context (45–51). They comprised child sex, child age, mothers’ age, number of children, mothers’ education, mothers’ employment, fathers’ education, fathers’ employment, rural/urban residence, polygynous household, household hunger, and socioeconomic status. In this study, socioeconomic status was assessed using a household asset score computed as the number of assets owned from a list of 39 items included in the survey administered to fathers (**Supplemental Table 2**). This was adapted from the socioeconomic questions included in the 2008 version of the Nigerian Demographic and Health Survey (DHS) tool (52). Household hunger was assessed using the Household Hunger Scale (53).

### Statistical analysis

Descriptive characteristics of participants were examined and are reported as means/percentages. The κ statistic and 95% CIs were used to examine the agreement between mothers’ and fathers’ reports of mothers’ decision-making autonomy as well as fathers’ complementary feeding support. The scale for the κ statistic was interpreted as 0–0.2 indicating poor agreement, 0.21–0.40 as fair agreement, 0.41–0.60 as moderate agreement, 0.61–0.80 as substantial agreement, and ≥0.81 as excellent agreement (54, 55). Due to the acceptable levels of agreement between mothers’ and fathers’ measurements of fathers’ complementary feeding support (percentage agreement ranged from 52.1% to 89.1%), only mothers’ measurement variables were used in the logistic regression and interaction models. The decision is also further supported by our qualitative findings from the same study (56), which indicated that the traditional roles of mothers as primary caregivers for children still prevail. Therefore, mothers’ perspectives can be more relevant in reflecting the types of support received.

Unadjusted and adjusted logistic regression analyses were then conducted to assess the associations of mothers’ decision-making autonomy with the outcome variables of minimum dietary diversity, minimum meal frequency, minimum acceptable diet, feeding of eggs, and feeding of fish. The key concepts for mothers’ autonomous household decision-making were tested in the logistic regression models using the composite scale and the subscales. Separate models were tested for each of the 5 outcome variables. Similarly, unadjusted and adjusted logistic regression analyses were also conducted to examine the association of fathers’ complementary feeding support with the 5 outcome variables. All associations were considered statistically significant at *P* < 0.05, and all analyses were adjusted for the covariates mentioned above.

Logistic regression interaction models were used to test whether the relation between mothers’ decision-making autonomy and optimal complementary feeding was stronger in mothers who reported high levels of complementary feeding support from fathers. Mothers’ decision-making autonomy was tested as a composite score and by analyzing the 2 dimensions of the scale as separate independent variables. The slope for effect of mothers’ decision-making autonomy on complementary feeding across levels of fathers’ complementary feeding support was predicted. As recommended, the predictor variables were not centered around the mean (57). Due to the exploratory nature of our analyses, multiple comparisons adjustment was not applied to the models during significance testing (58). Interactions were considered statistically significant at *P* < 0.1, and models tested were adjusted for covariate variables. Survey design was considered using Stata survey commands (svy) in version 16 (59).

## Results

The mean asset score for participant households was 10.4 assets; most households (90.1%) had low household hunger and very few (0.6%) had severe household hunger. There were also few households (21.1%) that were polygynous. On average participant households had 1.6 children, with an average age of 14.0 mo, and less than half (44.7%) of the children were female. Fathers were older than mothers, more educated (63.2% of fathers had some secondary education or higher compared with only 39.0% of mothers) and almost all fathers (98.4%) were employed, compared with only 55.2% of mothers being employed (**Table 1**). For the complementary feeding indicators, the proportion of children fed diets that met the minimum dietary diversity, minimum meal frequency, and minimum acceptable diet requirements were 64.7%, 72.6%, and 51.1%, respectively. In addition, 20.1% of children were fed eggs, and 43.5% of children were fed fish the previous day.

**Table 1:** Sociodemographic characteristics of study participants (*n* = 495)

Characteristic	Mean ± SE/percentage (*n*)
Mothers’ mean age, y	25.7 ± 0.3
Fathers’ mean age, y	36.6 ± 0.4
Mothers’ education: highest level of school completed (%)	
Never attended school	24.7 (122)
Primary	33.5 (164)
Secondary	33.2 (166)
Postsecondary	5.8 (32)
Fathers’ education: highest level of school completed (%)	
Never attended school	12.8 (61)
Primary	16.8 (80)
Secondary	42.2 (204)
Postsecondary	21.0 (104)
Mothers’ employment, % employed	55.2 (271)
Fathers’ employment, % employed	98.4 (480)

**Table 2** shows substantial to excellent agreement between mothers’ and fathers’ reports of mothers’ autonomous household decision-making for most domains of household decision-making, except for use of mothers’ cash earnings, which showed moderate agreement (57.6%, κ = 0.16). Feeding-related decisions and food-related purchases were the domains of decision-making most reported by mothers and fathers.

**Table 2:** Agreement in mothers’ and fathers’ reports on mothers’ autonomous household decision-making

Domains	Mothers’ measurements, %	Fathers’ measurements, %	Percentage agreement	κ
Food-related purchases	68.2	77.2	68.4	0.21
Feeding-related decisions	73.3	85.5	71.3	0.14
Household income and expenses	11.3	25.5	68.6	−0.02
Large household investments	9.7	18.2	76.0	0.02
Mothers’ ability to work outside the home	11.5	21.7	73.8	0.07
Use of father's cash earnings	11.7	21.0	72.7	0.02
Use of mother's cash earnings	63.3	48.5	57.6	0.16

There was substantial agreement between mothers’ and fathers’ reports about fathers’ overall involvement in complementary feeding (80.1%, κ = −0.04). **Table 3** shows that >80% of mothers and fathers reported that fathers supported child complementary feeding through the provision of money for food for the child. Very few mothers and fathers reported fathers providing “caregiving” complementary feeding support, such as helping with other chores so mother can prepare food or feed the child (ranged from 9.9% to 13.9%) or washing the child's hands before the child eats (ranged from 7.9% to 14.5%). There was substantial to excellent agreement between mothers’ reports of fathers’ support and fathers’ reports of their support, except for purchasing food specifically for the child, which showed moderate agreement (52.1%, κ = 0.07). A total of 42.0% of mothers reported receiving ≥1 type of support, and 0.4% reported receiving 6 types of support (**Supplemental Tables 3** and **4**).

**Table 3:** Agreement in mothers’ and fathers’ reports of complementary feeding support received from and provided by fathers

Support domains	Proportion of mothers who report receiving type of support, %	Proportion of fathers who report providing type of support, %	Percentage agreement	κ
Money for food for child	84.4	88.7	76.8	0.01
Purchases food specifically for child	29.3	52.9	52.1	0.07
Gives advice/reminds mother how to feed the child	21.2	12.9	77.2	0.20
Feeds the child himself	23.4	7.9	71.5	−0.03
Teaches child how to feed himself/herself	5.9	5.9	89.1	0.01
Washes child's hands before child eats	7.9	14.5	80.4	0.03
Helps with other chores so mother can prepare food or feed the child	13.9	9.9	77.8	−0.05

In the adjusted models, higher scores on the “Food and feeding related” subscale of autonomous decision-making were associated with minimum meal frequency, minimum acceptable diet, and feeding fish on the previous day (**Supplemental Table 5**). Higher scores on the “Household finance related” subscale of autonomous decision-making were associated with feeding fish the previous day in adjusted models (Supplemental Table 5). Unadjusted (**Supplemental Table 6**) and adjusted associations (**Table 4**) between the composite scale for mothers’ autonomous household decision-making and the complementary feeding indicators show that higher scores on the composite scale were associated with minimum dietary diversity [adjusted odds ratio (AOR): 1.2; 95% CI, 1.0, 1.3)], minimum meal frequency (AOR, 1.3; 95% CI, 1.1, 1.5), minimum acceptable diet (AOR, 1.2; 95% CI, 1.1, 1.4), and feeding fish (AOR, 1.2; 95% CI, 1.1, 1.4). The logistic regression results suggest that putting both subscales together as a composite scale showed positive associations with more complementary feeding indicators than using the subscales separately. As a result, only the composite scale was used in the interaction models with fathers’ complementary feeding support.

**Table 4:** Adjusted associations of mothers’ autonomous household decision-making and fathers’ complementary feeding support with minimum dietary diversity, minimum meal frequency, minimum acceptable diet, feeding of eggs, and feeding of fish^1^

Independent variables^2^	Minimum dietary diversity	Minimum meal frequency	Minimum acceptable diet	Feeding of eggs	Feeding of fish
Mothers’ autonomous household decision-making	1.2 (1.0, 1.3)*	1.3 (1.1, 1.5)**	1.2 (1.1, 1.4)**	0.9 (0.8, 1.1)	1.2 (1.1, 1.4)**
Fathers’ complementary feeding support	1.4 (1.1, 1.8)*	1.3 (1.1, 1.7)*	1.4 (1.1, 1.7)**	1.5 (1.2, 1.9)**	1.3 (1.1, 1.6)*

1 Models adjusted for child sex, child age, mothers’ age, number of children, mothers’ education, fathers’ education, mothers’ employment, fathers’ employment, rural/urban residence, polygynous household, household hunger, and socioeconomic status. Values are ORs (95% CI). ^*,**^Denotes significant association; *P < 0.05; **P < 0.001.

2 Variables are summative scores of item responses in the final scales.

**Figure 1:**
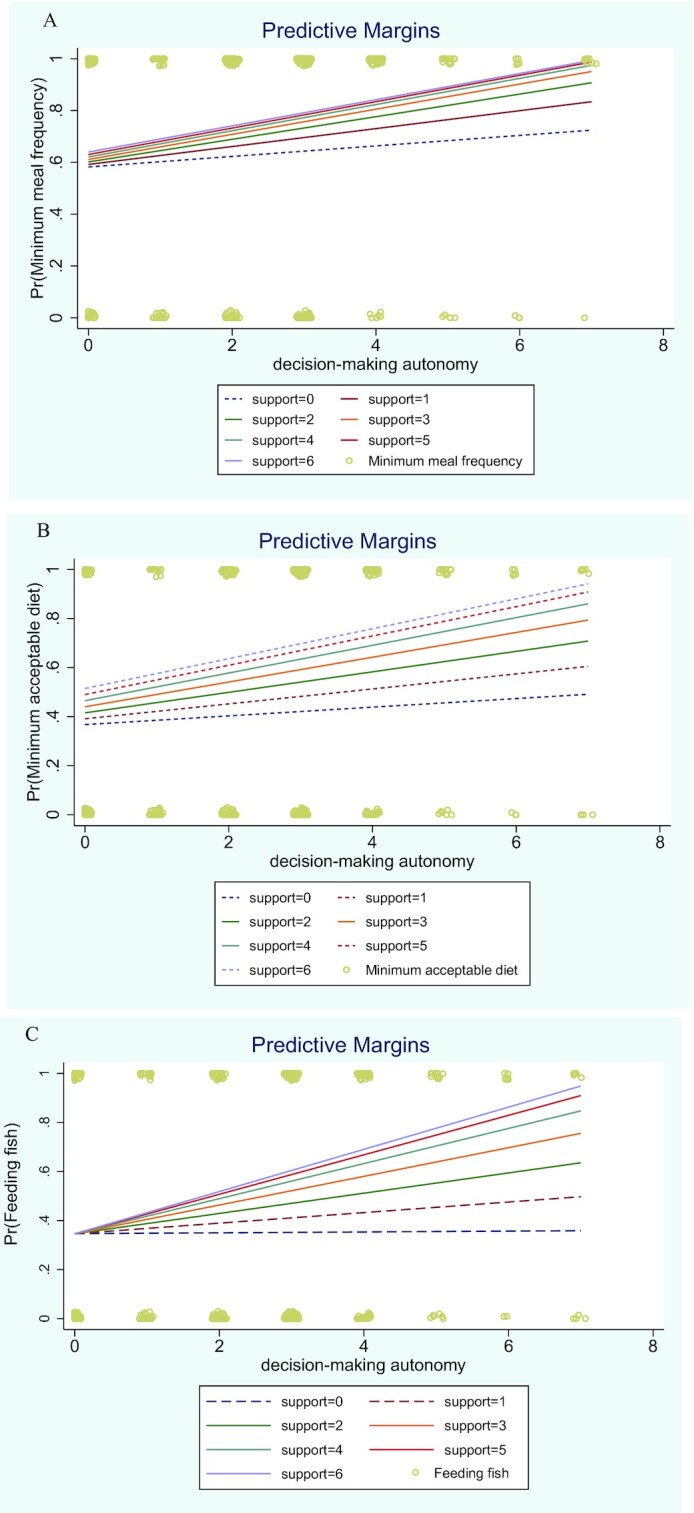
(A) Margins plots of the adjusted predicted probabilities for meeting the minimum meal frequency indicator requirements for mothers’ autonomous household decision-making across varying levels of fathers’ complementary feeding support. (B) Margins plots of the adjusted predicted probabilities for meeting the minimum acceptable diet indicator requirements for mothers’ autonomous household decision-making across varying levels of fathers’ complementary feeding support. (C) Margins plots of the adjusted predicted probabilities for feeding young children fish the previous day for mothers’ autonomous household decision-making across varying levels of fathers’ complementary feeding support. Pr, probability.

In the unadjusted (**Supplemental Table 7**A–J) and adjusted (margins plots, **Figure 1**A–C) moderation models using the composite decision-making scale, the simple slopes for decision-making were significant for values of ≥2 reported support actions for minimum meal frequency, minimum acceptable diet indicator, and feeding fish the previous day. There were no significant moderation effects for the minimum dietary diversity indicator.

With respect to fathers’ complementary feeding support, the logistic regression results show that higher scores on the support scale were associated with all the complementary feeding indicators after controlling for covariates: minimum dietary diversity (AOR: 1.4; 95% CI: 1.1, 1.8), minimum meal frequency (AOR: 1.3; 95% CI: 1.1, 1.7), minimum acceptable diet (AOR: 1.4; 95% CI: 1.1, 1.7), feeding of eggs (AOR: 1.5; 95% CI: 1.2, 1.9), and feeding of fish (AOR: 1.3; 95% CI: 1.1, 1.6) (Table 4).

## Discussion

The key finding from this study is that high complementary feeding support from fathers (≥2 reported actions) strengthened the association of mothers’ autonomous household decision-making with minimum meal frequency, minimum acceptable diet, and feeding fish the previous day. These findings imply a synergistic interaction effect of fathers’ complementary feeding support on the relation between mothers’ autonomy in household decision-making and minimum meal frequency, minimum acceptable diet, and feeding children fish the previous day (60). However, a synergistic relation was not observed for the minimum dietary diversity indicator, despite it being a component of the minimum acceptable diet indicator. Our divergent findings for the minimum dietary diversity indicator highlight the importance of examining the dimensions of autonomy and the concordance between mothers’ and fathers’ perspectives of mothers’ autonomy in household decision-making and fathers’ complementary feeding support.

Previous studies in low- and middle-income countries defined autonomy as a multidimensional construct with components that include “child-related decision-making” (33, 43, 61). A systematic review of women's autonomy and child nutritional status also noted that mothers might not have high autonomy in all dimensions measured (62). In this study, 68.2% to 73.3% of mothers, and 77.2% to 85.5% of fathers, reported that mothers had autonomy in household decisions related to food and feeding. Also, 9.7% to 11.7% of mothers, and 18.2% to 25.5% of fathers, reported that mothers had autonomy in financial household decisions, such as income and expenses and large household investments. The κ statistics showed substantial to excellent agreement between mothers’ and fathers’ reports of mothers’ autonomy for these domains of decision-making. This suggests that in Kaduna, both mothers and fathers agree that most mothers in Kaduna have more autonomy in making food and feeding decisions but less autonomy in making financial decisions.

The nonsignificant finding for the minimum dietary diversity indicator in the moderation models suggests that mothers’ lack of autonomy in financial decision-making could be translating into constraints in acquiring diverse and nutritious foods for their young children, as has been reported in other studies (63, 64). Therefore, complementary feeding social support interventions could employ strategies that improve mothers’ autonomy in household financial decisions to further improve feeding children healthy, diversified diets that consist of nutrient-dense foods.

In this study, we also found that 84.4% of mothers and 88.7% of fathers reported that fathers support complementary feeding by providing money for food, and 13.9% of mothers and 9.9% of fathers reported that fathers support complementary feeding by helping with household chores so that mothers can prepare food or feed the child. Both domains of complementary feeding support had substantial agreement between mothers’ and fathers’ reports, suggesting that most fathers in Kaduna support complementary feeding mainly by providing money for food, with less provision of “caregiving support.” Fathers’ roles as “providers or suppliers” have been documented in several studies as helpful in improving availability of diverse complementary foods (8, 17, 18, 65, 66). A previous qualitative article on household gender roles and paternal/maternal involvement in complementary feeding from this same study in Kaduna showed that fathers’ perspectives of their traditional roles as “providers” influence their involvement and support for complementary feeding (56). The similarities in both the qualitative and quantitative findings suggest that future SBCC interventions in Kaduna could leverage the traditional roles of fathers as “providers” to continue their financial support for complementary feeding so mothers can exercise their autonomy to purchase and feed recommended complementary foods to children.

In the logistic regression models, there were significant but small positive associations between complementary feeding support and all the complementary feeding indicators. The positive associations between complementary feeding support and the complementary feeding indicators are similar to findings reported in other studies in sub-Saharan Africa (8). The small magnitude of the effect sizes is likely because most fathers provide only 1 type of support, which although necessary, might not produce as much of an effect on complementary feeding practices as a variety of types of support. However, we did not test this assumption in this study. Nevertheless, our findings reflect the need to encourage fathers in Kaduna to progress beyond providing money for food by adding other kinds of support (e.g., participating in food preparation, household chores), which allow mothers to have more time to prepare and feed recommended foods to young children (18, 22, 67).

The complementary feeding indicators reported in this study are higher than the reported indicators in the Nigerian 2018 DHS. The participants from our study were sampled to represent the Igabi LGA. Approximately 60% of study participants lived in the urban areas of Igabi whereas in the 2018 DHS, participants were sampled to represent the state of Kaduna and included ∼46% in urban areas (26). Complementary feeding indicators are often higher in urban compared with rural areas (68).

This study adds to the complementary feeding evidence base in 2 ways. To the best of our knowledge, our study is the first to examine the moderating effect of fathers’ complementary feeding support on the association of mothers’ autonomy in household decision-making with complementary feeding indicators. Our use of Cohen κ to document the concordance between mothers’ and fathers’ perspectives of fathers’ complementary feeding support and mothers’ autonomous household decision-making is another strength of our study.

This study's limitations include our use of cross-sectional data, which limits our ability to draw causal inferences and to establish temporality of the relations between our independent variables of interest (mothers’ autonomous household decision-making and fathers’ complementary feeding support) and the complementary feeding indicators. Although previous research on these factors does not suggest reverse relations, we were unable to completely rule out the plausibility of reverse associations. Another limitation of our study is that the reported perspectives of the parents about mothers’ decision-making autonomy and fathers’ complementary feeding support could be influenced by social desirability bias. However, the high level of concordance in the parents’ reports suggests this is less of a concern. Furthermore, although having cohabiting parents as participants in the study was helpful in examining the concordance in their reported perspectives, this eligibility criterion limited the applicability of this study's findings to noncohabiting parents in Kaduna. Future complementary feeding social support and/or autonomy intervention research in Kaduna could include perspectives from noncohabiting parents to extend the evidence from this research. Related to this, this study also did not include data collection from other family members such as grandmothers and other influential female family members who influence complementary feeding, decision-making autonomy, and complementary feeding social support within the Nigerian context (69, 70). Future complementary feeding SBCC interventions should use a family systems approach to understand how support from other influential family members influences these relations (71).

**Figure 2:**
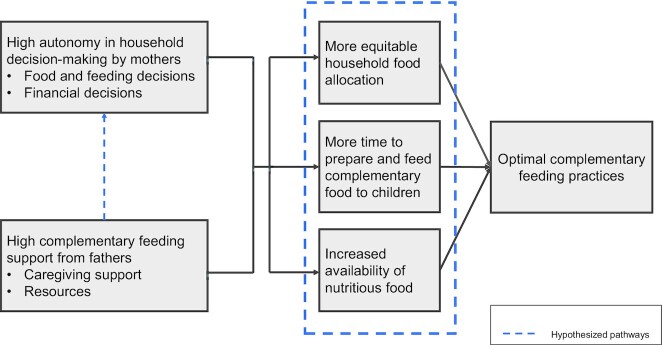
Proposed pathways through which father's complementary feeding support can enhance the positive impact of mother's decision-making autonomy on complementary feeding practices.

In conclusion, our study demonstrated that higher levels of maternal autonomy in household decision-making in combination with high levels of complementary feeding support from fathers are associated with meeting the requirements for minimum meal frequency, minimum acceptable diet, and feeding children fish in Nigeria. This study further demonstrated that there was moderate to substantial agreement between mothers’ and fathers’ reports of mothers’ autonomous decision-making in the household, and moderate to excellent agreement between mothers’ and fathers’ reports of fathers’ complementary feeding support. Explaining the linkages through which fathers’ complementary feeding support strengthens the positive effects of mothers’ decision-making autonomy on complementary feeding practices is important. There are varied potential pathways including increased availability of nutritious foods, more equitable intrahousehold food allocation, and increased time availability for complementary feeding (**Figure 2**). Future research can be helpful in testing these pathways. The findings from this research suggest that complementary feeding social and behavior change interventions in Nigeria need to be more gender-transformative in their approach, utilizing strategies that address both the gendered inequities in decision-making and fathers’ participation in caregiving tasks associated with complementary feeding practices, to have larger effects in improving complementary feeding practices.

## Supplementary Material

nzac098_Supplemental_File

## Data Availability

The data that support the findings of this study are available from the corresponding author upon request after receiving approval from Alive & Thrive.

## Notes

This research was funded by the Alive & Thrive initiative, managed by FHI Solutions, and funded by the Bill & Melinda Gates Foundation, Irish Aid, and other donors under grant numbers INV-029432 and OPP 1135932.

Author disclosures: SK and SB are employed by Alive & Thrive. They were involved in the implementation and evaluation of the intervention in Nigeria, but not in data collection or analysis for this study. All other authors report no conflicts of interest. SLM served as Guest Editor for the Journal but played no role in the review or acceptance of this manuscript.

Supplemental Tables 1–7 and Supplemental Materials are available from the “Supplementary data” link in the online posting of the article and from the same link in the online table of contents at https://academic.oup.com/cdn/.

Abbreviations used: AOR, adjusted odds ratio; CBO, community-based organization; CHEW, community health extension worker; DHS, Demographic and Health Survey; EFA, exploratory factor analysis; ICARE, I Care Women and Youth Initiative; IYCF, infant and young child feeding; KMO test, Kaiser–Meyer–Olkin test; LGA, local government area; ODK, Open Data Kit; Pr, probability; SBCC, social and behavior change communication.
