# Supplementary material for: Fathers’ Complementary Feeding Support Strengthens the Association Between Mothers’ Decision-Making Autonomy and Optimal Complementary Feeding in Nigeria
Source: Curr Dev Nutr. 2022 Jun 2;6(7):nzac098. doi: 10.1093/cdn/nzac098 (PMC9283102; doi:10.1093/cdn/nzac098)
Supplement: nzac098_Supplemental_File [file nzac098_supplemental_file.docx]

**Supplemental Table 1**: Differences for mothers and fathers in hypothesized dimensionality and internal consistency of the scale for mothers’ decision-making autonomy.

| Construct | **Mothers’ measurements** | | |  | **Fathers’ measurements** | | |  |
| --- | --- | --- | --- | --- | --- | --- | --- | --- |
|  | Dimensionality | Final items in the scale | Cronbach’s alpha | Inter-item covariance | Dimensionality | Final items in the scale | Cronbach’s alpha | Inter-item covariance |
| Mothers’ decision-making autonomy | Bidimensional (2 factors retained; Factor 1 explains 74% of variance in the scale). | **Factor 1 has 3 items**: food-related purchases; feeding related decisions; use of mother’s cash earnings. | 0.81 | 0.127 | Unidimensional (1 factor retained. Factor explains 88% of variance in the scale). | **Single factor which has 7 items**: Food-related purchases; Feeding related decisions; Use of mother’s cash earnings; Household weekly/monthly income; Large household investments; Mother’s ability to work outside the home; Use of father’s cash earnings. | 0.75 | 0.053 |
| Mothers’ decision-making autonomy | Bidimensional (2 factors retained; Factor 1 explains 74% of variance in the scale). | **Factor 2 has 4 items**: household weekly/monthly income; large household investments; mother’s ability to work outside the home; use of father’s cash earnings. | 0.69 | 0.035 |  |  |  |  |

**Supplemental Table 2**: Potential assets of participant households

| Asset | Asset Code |
| --- | --- |
| Car/truck/van | 1 |
| Motorbike | 2 |
| Household electric generator | 3 |
| Telephone/mobile phone | 4 |
| Sewing/weaving/embroidering machine | 5 |
| Video player | 6 |
| TV | 7 |
| CD player | 8 |
| Desktop computer | 9 |
| Laptop computer | 10 |
| Printer (for computer) | 11 |
| Camera | 12 |
| Refrigerator | 13 |
| Freezer | 14 |
| Air conditioner | 15 |
| Washing machine | 16 |
| Clothes drying machine | 17 |
| Water heater | 18 |
| Oven | 19 |
| Gas cooker | 20 |
| Electric cooker | 21 |
| Watch | 22 |
| Vacuum cleaner | 23 |
| Dehumidifier | 24 |
| Microwave oven | 25 |
| Bed/mattress | 26 |
| Sofa | 27 |
| Tables and chairs | 28 |
| Electric fan (ceiling, table, wall) | 29 |
| Bicycle | 30 |
| Motored boat | 31 |
| Water pumping machine | 32 |
| Grain milling machine | 33 |
| Horse/Ox Cart | 34 |
| Pig | 35 |
| Cow/bulls | 36 |
| Goats/sheep | 37 |
| Poultry (Chicken/duck/geese) | 38 |
| Electricity | 39 |

**Supplemental Table 3:** Distribution of reported support scores using mothers’ measurements

| **Support score** | **Frequency** | **Percentage** | **Cumulative percentage** |
| --- | --- | --- | --- |
| 0 | 7 | 1.41 | 1.41 |
| 1 | 209 | 42.22 | 43.64 |
| 2 | 162 | 32.73 | 76.36 |
| 3 | 88 | 17.78 | 94.14 |
| 4 | 23 | 4.65 | 98.79 |
| 5 | 4 | 0.81 | 99.6 |
| 6 | 2 | 0.4 | 100 |
| Total | 495 | 100 |  |

**Supplemental Table 4**: Scale properties and range of reported scores for mothers’ autonomous household decision-making and fathers’ complementary feeding support scales.

| Scale | Final items in scale | Range of reported scores |
| --- | --- | --- |
| Mothers’ autonomous decision-making |  |  |
| Composite scale | 7 items | 0-6 |
| Sub-scale 1: Food and feeding-related | 3 items | 0-3 |
| Sub-scale 2: Household finance-related | 4 items | 0-4 |
| Fathers’ complementary feeding support | 7 items | 0-7 |

**Supplemental Table 5** Adjusted^+^ associations of the composite and sub-scales of mothers’ autonomous household decision-making with the complementary feeding indicators^b^.

| **Mothers’ autonomous household decision-making^c^** | **Minimum dietary diversity** | **Minimum meal frequency** | **Minimum acceptable diet** | **Feeding of eggs** | **Feeding of fish** |
| --- | --- | --- | --- | --- | --- |
| Composite scale | 1.2 (1.0, 1.3) * | 1.3 (1.1, 1.5) ** | 1.2 (1.1, 1.4) ** | 0.9 (0.8, 1.1) | 1.2 (1.1, 1.4) ** |
| Sub-scale 1: Food and feeding related | 1.2 (1.0, 1.37) | 1.4 (1.2, 1.7) ** | 1.3 (1.1, 1.5) ** | 0.9 (0.7, 1.1) | 1.2 (1.0, 1.4) * |
| Sub-scale 2: Household finance related | 1.2 (0.9, 1.6) | 1.3 (0.9, 1.7) | 1.2 (1.0, 1.6) | 0.9 (0.7, 1.2) | 1.5 (1.2, 1.8) * |

*+Models adjusted for child sex, child age, mothers’ age, number of children, mothers’ education, fathers’ education, mothers’ employment, fathers’ employment, rural/urban residence, polygynous household, household hunger and socio-economic status.*

*bValues are Odds ratios (95% Confidence Interval)         * p<0.05              **p<0.001*

*cVariables* *are summative scores obtained from summing the item responses in the composite and sub-scales of decision-making autonomy*

**Supplemental Table 6** Unadjusted associations between mothers’ autonomous household decision-making (composite scale), fathers’ complementary feeding support, and minimum dietary diversity, minimum meal frequency, minimum acceptable diet, feeding of eggs and feeding of fish^b^.

| Independent variables^b^ | **Minimum dietary diversity** | **Minimum meal frequency** | **Minimum acceptable diet** | **Feeding of eggs** | **Feeding of fish** |
| --- | --- | --- | --- | --- | --- |
|  | **OR (95% CI)** | **OR (95% CI)** | **OR (95% CI)** | **OR (95% CI)** | **OR (95% CI)** |
| Mothers’ autonomous household decision-making | 1.1 (1.0, 1.3) | 1.3 (1.1, 1.5) ** | 1.2 (1.1, 1.4) ** | 0.9 (0.8, 1.0) | 1.2 (1.1, 1.3) ** |
| Fathers’ complementary feeding support | 1.5 (1.2, 1.9) ** | 1.3 (1.0, 1.6) * | 1.5 (1.2, 1.8) ** | 1.5 (1.2, 1.9) ** | 1.4 (1.2, 1.7) ** |

*^a^Values are Odds ratios (95% Confidence Interval) * p<0.05 **p<0.001*

*^b^Variables* *are summative scores obtained from summing the item responses in the final scales.*

**Supplemental Tables 7a**: Simple slopes of the effect of mothers’ autonomous household decision-making on Minimum dietary diversity at varying levels of fathers’ complementary feeding support (Unadjusted).

|  | Minimum dietary diversity-Unadjusted | | | | | |
| --- | --- | --- | --- | --- | --- | --- |
| Decision-making X Support | dy/dx | Std. Err. | t | P>t | [95% C.I] | |
| 0 | -0.04 | 0.03 | -1.11 | 0.27 | -0.11 | 0.03 |
| 1 | -0.03 | 0.02 | -0.16 | 0.87 | -0.04 | 0.04 |
| 2 | 0.03 | 0.01 | 1.96 | 0.05* | 0.00 | 0.06 |
| 3 | 0.05 | 0.02 | 2.48 | 0.01* | 0.01 | 0.09 |
| 6 | 0.06 | 0.03 | 2.30 | 0.02* | 0.01 | 0.12 |
| 5 | 0.07 | 0.03 | 2.02 | 0.04* | 0.00 | 0.14 |
| 6 | 0.07 | 0.04 | 1.74 | 0.08* | -0.01 | 0.16 |

**Supplemental Tables 7b:** Simple slopes of the effect of mothers’ autonomous household decision-making on Minimum dietary diversity at varying levels of fathers’ complementary feeding support (Adjusted).

| Minimum dietary diversity-Adjusted | | | | | | |
| --- | --- | --- | --- | --- | --- | --- |
| Decision-making X Support | dy/dx | Std. Err. | t | P>t | [95% C.I] | |
| 0 | -0.01 | 0.03 | -0.18 | 0.86 | -0.09 | 0.04 |
| 1 | 0.01 | 0.02 | 0.37 | 0.71 | -0.04 | 0.04 |
| 2 | 0.02 | 0.01 | 1.48 | 0.14 | -0.01 | 0.05 |
| 3 | 0.03 | 0.02 | 1.57 | 0.12 | 0.00 | 0.08 |
| 4 | 0.04 | 0.03 | 1.38 | 0.17 | 0.00 | 0.11 |
| 5 | 0.05 | 0.04 | 1.26 | 0.21 | -0.01 | 0.14 |
| 6 | 0.05 | 0.04 | 1.17 | 0.24 | -0.01 | 0.17 |

**Supplemental Tables 7c**: Simple slopes of the effect of mothers’ autonomous household decision-making on Minimum meal frequency at varying levels of fathers’ complementary feeding support (Unadjusted)

|  | Minimum meal frequency-Unadjusted | | | | | |
| --- | --- | --- | --- | --- | --- | --- |
| Decision-making X Support | dy/dx | Std. Err. | t | P>t | [95% C.I] | |
| 0 | 0.03 | 0.03 | 0.78 | 0.43 | -0.04 | 0.09 |
| 1 | 0.04 | 0.02 | 1.89 | 0.06* | 0.00 | 0.07 |
| 2 | 0.04 | 0.01 | 3.27 | <0.01* | 0.02 | 0.07 |
| 3 | 0.05 | 0.02 | 2.70 | 0.01* | 0.01 | 0.09 |
| 4 | 0.05 | 0.03 | 2.04 | 0.04* | 0.00 | 0.10 |
| 5 | 0.06 | 0.03 | 1.65 | 0.10* | -0.01 | 0.12 |
| 6 | 0.06 | 0.04 | 1.39 | 0.16 | -0.02 | 0.14 |

**Supplemental Tables 7d**: Simple slopes of the effect of mothers’ autonomous household decision-making on Minimum meal frequency at varying levels of fathers’ complementary feeding support (Adjusted).

|  | Minimum meal frequency-Adjusted | | | | | |
| --- | --- | --- | --- | --- | --- | --- |
| Decision-making X Support | dy/dx | Std. Err. | t | P>t | [95% C.I] | |
| 0 | 0.02 | 0.04 | 0.59 | 0.60 | -0.05 | 0.09 |
| 1 | 0.04 | 0.02 | 1.93 | 0.05* | -0.00 | 0.08 |
| 2 | 0.05 | 0.01 | 3.65 | <0.01* | 0.02 | 0.08 |
| 3 | 0.06 | 0.02 | 3.06 | <0.01* | 0.02 | 0.10 |
| 4 | 0.06 | 0.03 | 2.38 | 0.02* | 0.01 | 0.12 |
| 5 | 0.07 | 0.04 | 1.94 | 0.05* | -0.00 | 0.14 |
| 6 | 0.07 | 0.04 | 1.63 | 0.10* | -0.01 | 0.15 |

**Supplemental Tables 7e**: Simple slopes of the effect of mothers’ autonomous household decision-making on Minimum acceptable diet at varying levels of fathers’ complementary feeding support (Unadjusted).

|  | Minimum acceptable diet-Unadjusted | | | | | |
| --- | --- | --- | --- | --- | --- | --- |
| Decision-making X Support | dy/dx | Std. Err. | t | P>t | [95% C.I] | |
| 0 | -0.00 | 0.03 | -0.14 | 0.89 | -0.07 | 0.06 |
| 1 | 0.02 | 0.02 | 1.01 | 0.31 | -0.02 | 0.06 |
| 2 | 0.05 | 0.01 | 3.14 | <0.01* | 0.02 | 0.08 |
| 3 | 0.07 | 0.02 | 3.12 | <0.01* | 0.03 | 0.11 |
| 4 | 0.08 | 0.03 | 2.72 | 0.01* | 0.02 | 0.14 |
| 5 | 0.09 | 0.04 | 2.41 | 0.02* | 0.02 | 0.16 |
| 6 | 0.09 | 0.04 | 2.11 | 0.04* | 0.01 | 0.18 |

**Supplemental Tables 7f**: Simple slopes of the effect of mothers’ autonomous household decision-making on Minimum acceptable diet at varying levels of fathers’ complementary feeding support (Adjusted).

|  | Minimum acceptable diet-Adjusted | | | | | |
| --- | --- | --- | --- | --- | --- | --- |
| Decision-making X Support | dy/dx | Std. Err. | t | P>t | [95% C.I] | |
| 0 | 0.02 | 0.03 | 0.52 | 0.60 | -0.05 | 0.08 |
| 1 | 0.03 | 0.02 | 1.51 | 0.13 | -0.01 | 0.07 |
| 2 | 0.04 | 0.01 | 2.93 | <0.01* | 0.01 | 0.07 |
| 3 | 0.05 | 0.02 | 2.42 | 0.02* | 0.01 | 0.10 |
| 4 | 0.06 | 0.03 | 1.90 | 0.06* | -0.00 | 0.13 |
| 5 | 0.07 | 0.04 | 1.62 | 0.11 | -0.01 | 0.15 |
| 6 | 0.07 | 0.05 | 1.44 | 0.15 | -0.03 | 0.17 |

**Supplemental Tables 7g**: Simple slopes of the effect of mothers’ autonomous household decision-making on Feeding of eggs at varying levels of fathers’ complementary feeding support (Unadjusted).

|  | Feeding of eggs-Unadjusted | | | | | |
| --- | --- | --- | --- | --- | --- | --- |
| Decision-making X Support | dy/dx | Std. Err. | t | P>t | [95% C.I] | |
| 0 | -0.03 | 0.02 | -1.55 | 0.12 | -0.07 | 0.01 |
| 1 | -0.03 | 0.02 | -1.83 | 0.07* | -0.06 | 0.00 |
| 2 | -0.02 | 0.01 | -1.67 | 0.10* | -0.05 | 0.00 |
| 3 | -0.01 | 0.02 | -0.52 | 0.60 | -0.05 | 0.03 |
| 4 | 0.01 | 0.04 | 0.17 | 0.87 | -0.07 | 0.08 |
| 5 | 0.03 | 0.06 | 0.47 | 0.64 | -0.09 | 0.14 |
| 6 | 0.05 | 0.07 | 0.65 | 0.52 | -0.10 | 0.19 |

**Supplemental Tables 7h**: Simple slopes of the effect of mothers’ autonomous household decision-making on Feeding of eggs at varying levels of fathers’ complementary feeding support (Adjusted)

|  | Feeding of eggs-Adjusted | | | | | |
| --- | --- | --- | --- | --- | --- | --- |
| Decision-making X Support | dy/dx | Std. Err. | t | P>t | [95% C.I] | |
| 0 | -0.03 | 0.02 | -1.37 | 0.17 | -0.07 | 0.01 |
| 1 | -0.02 | 0.01 | -1.82 | 0.07* | -0.05 | 0.00 |
| 2 | -0.02 | 0.01 | -1.86 | 0.06* | -0.04 | 0.00 |
| 3 | -0.02 | 0.02 | -0.84 | 0.40 | -0.06 | 0.02 |
| 4 | -0.01 | 0.04 | -0.29 | 0.77 | -0.08 | 0.06 |
| 5 | -0.00 | 0.05 | -0.03 | 0.97 | -0.11 | 0.10 |
| 6 | 0.01 | 0.07 | 0.11 | 0.91 | -0.13 | 0.15 |

**Supplemental Tables 7i**: Simple slopes of the effect of mothers’ autonomous household decision-making on Feeding of fish at varying levels of fathers’ complementary feeding support (Unadjusted).

|  | Feeding of fish-Unadjusted | | | | | |
| --- | --- | --- | --- | --- | --- | --- |
| Decision-making X Support | dy/dx | Std. Err. | t | P>t | [95% C.I] | |
| 0 | -0.01 | 0.03 | -0.40 | 0.69 | -0.07 | 0.05 |
| 1 | 0.01 | 0.02 | 0.67 | 0.50 | -0.03 | 0.05 |
| 2 | 0.04 | 0.01 | 2.88 | <0.01* | 0.01 | 0.07 |
| 3 | 0.07 | 0.02 | 3.23 | <0.01* | 0.03 | 0.11 |
| 4 | 0.09 | 0.03 | 2.96 | <0.01* | 0.03 | 0.15 |
| 5 | 0.10 | 0.04 | 2.71 | 0.01* | 0.03 | 0.17 |
| 6 | 0.10 | 0.04 | 2.45 | 0.02* | 0.02 | 0.19 |

**Supplemental Tables 7j**: Simple slopes of the effect of mothers’ autonomous household decision-making on Feeding of fish at varying levels of fathers’ complementary feeding support (Adjusted)

|  | Feeding of fish-Adjusted | | | | | |
| --- | --- | --- | --- | --- | --- | --- |
| Decision-making X Support | dy/dx | Std. Err. | t | P>t | [95% C.I] | |
| 0 | 0.00 | 0.03 | 0.06 | 0.96 | -0.06 | 0.06 |
| 1 | 0.02 | 0.02 | 1.11 | 0.27 | -0.02 | 0.06 |
| 2 | 0.04 | 0.01 | 2.92 | <0.01* | 0.01 | 0.07 |
| 3 | 0.06 | 0.02 | 2.76 | <0.01* | 0.02 | 0.10 |
| 4 | 0.08 | 0.03 | 2.39 | 0.02* | 0.01 | 0.14 |
| 5 | 0.09 | 0.04 | 2.21 | 0.03* | 0.01 | 0.17 |
| 6 | 0.10 | 0.05 | 2.10 | 0.04* | 0.01 | 0.20 |

**Date Collection Tools: Mothers’ Questionnaire**

# MODULE 2: INFANT AND YOUNG CHILD FEEDING PRACTICES

Now I’d like to ask you some questions about how you have fed (NAME).

| **No.** | **Questions** | | **Codes** | | |  |
| --- | --- | --- | --- | --- | --- | --- |
|  | Is (NAME) breastfeeding? | | Yes 1**>>**204  No 0  Refused 999 | | |  |
|  | For how long did you breastfeed (NAME)?  IF LESS THAN ONE MONTH, RECORD 0. | | Months ___________  Never breastfed………………………………….98>>205  Don’t Know/cannot remember 99  Refused 999 | | |  |
|  | Why did you stop breastfeeding?  Any other reasons?  DO NOT READ RESPONSES.  ***(MULTIPLE RESPONSES POSSIBLE)***  **AFTER OBTAINING RESPONSE, SKIP TO Q205.** | | Problems with breast (pain) 1  Child did not suck well 2  Child not growing well 3  Not enough time to feed child 4  Child already grown up/ No need for breastfeeding 5  Mother got pregnant 6  New baby born 7  Cracked nipples 8  Felt not enough breastmilk 9  Other (specify)______________ 98  Don’t know 99  Refused 999 | | |  |
|  | How many times did you breastfeed (NAME) yesterday, during the day and night? | | Number of times ___ ___  Don’t know 99  Refused 999 | | |  |
|  | Yesterday during the day or night, did (NAME) drink any **(**LIQUID ITEMS***)***?  READ THE LIST OF LIQUIDS, STARTING WITH BREAST MILK. | | | | | |
|  | **ITEM** | **RESPONSE** | | | **CODES** | |
|  | 1. Breast milk |  | | | Yes 1  No 0  Don’t know 99  Refused..............999 | |
|  | 2. Water |  | | |  |  |
|  | 3. Baby formula (NAN, SMA Gold, SMA Progress, Peak 123) |  | | |  |  |
|  | 4. Any other kind of milk (powdered or fresh cow/goat milk) |  | | |  |  |
|  | 5. Yogurt |  | | |  |  |
|  | 6. Fruit juice (made at home) |  | | |  |  |
|  | 7. Fruit juice (purchased, packaged) |  | | |  |  |
|  | 8. Water-based liquids, teas, sugar water, coffee |  | | |  |  |
|  | 9. Clear broth (liquid from cooking meat, fish, or vegetables) |  | | |  |  |
|  | 10. Soda (Coke, Fanta, Sprite, orange squash, etc.) |  | | |  |  |
|  | IF OPTIONS 3, 4, OR 5 ARE CHOSEN FOR Q205, THEN ASK Q206. OTHERWISE GO TO Q207. | | | | | |
|  | Other than breast milk, how many times did (NAME) drink other milk, formula or yogurt yesterday, during the day and night?  DO NOT INCLUDE NUMBER OF TIMES THE CHILD WAS BREASTFED IN THIS QUESTION. THIS VARIABLE IS ONLY TO CAPTURE MILK OR MILK PRODUCTS **OTHER THAN BREAST MILK**. | | | Number of times ___ ___  Not given yet 88  Don’t know 99  Refused 999 | | |
|  | Please describe everything that (NAME) ate yesterday during the day or night, whether at home or outside the home.  Starting from the morning, what was the first thing (NAME) ate after waking up?  Please tell me everything (NAME)  ate at that time. *Probe:*Anything else? U*nti*l *respondent says nothing e*l*se.*  ASK ABOUT THE CHILD’S MEALS AND SNACKS THROUGHOUT THE DAY UNTIL RESPONDENT SAYS THE CHILD WENT TO SLEEP FOR THE NIGHT.  PROBE IF MIXED DISH: What ingredients were in the dish? Anything else?  MARK FOOD GROUPS INCLUDED IN ALL DISHES THE CHILD ATE YESTERDAY, INLCUDING ALL ITEMS IN MIXED DISHES. ONCE THE RESPONDENT FINISHES NAMING FOODS, READ EACH RESPONSE THAT WAS NOT MENTIONED. ASK: | | | | | |

| NO | TIME | DISH | INGREDIENTS |
| --- | --- | --- | --- |
| 1. | Breakfast | e.g. Porridge | Maize, soya, sugar, milk |
|  |  |  |  |
|  |  |  |  |
|  |  |  |  |
| 2. | Between Breakfast and Lunch | e.g. Banana | Banana |
|  |  |  |  |
|  |  |  |  |
|  |  |  |  |
| 3. | Lunch | e.g Egusi soup | Onion, palm oil, egusi, fish, pumpkin leaf, salt |
|  |  |  |  |
|  |  |  |  |
|  |  |  |  |
| 4. | Between Lunch and Dinner |  |  |
|  |  |  |  |
|  |  |  |  |
| 5. | Dinner |  |  |
|  |  |  |  |
|  |  |  |  |

|  | AFTER COMPLETING THE DIETARY RECALL IN QUESTION 207, USE THE INFORMATION TO FILL OUT THE TABLE BELOW. | | | | |
| --- | --- | --- | --- | --- | --- |
|  | ITEM | RESPONSE | | | Yes 1  No 0 |
|  | 1. Purchased baby cereals (Golden Morn, Frisco Cream, Frisco Progress, Quaker Oats) |  | | |  |
|  | 2. Cereals (rice, maize, noodles, wheat, millet, sorghum, wheat) |  | | |  |
|  | 3. Legumes (beans, soya, groundnuts, peas, pigeon peas, moimoi, akara, lentils) |  | | |  |
|  | 4. Green leafy vegetables (spinach, pumpkin leaf, cassava leaf, etc.) |  | | |  |
|  | 5. Pumpkin, yellow sweet potatoes, carrots |  | | |  |
|  | 6. Irish/white potatoes, white yams, cocoyams, cassava, other white roots, or plantain |  | | |  |
|  | 7. Ripe papaya or mango or palm nuts |  | | |  |
|  | 8. Any other fruits such as oranges, banana, apple, watermelon, guava |  | | |  |
|  | 9. Any other vegetables (eggplant, okra, onions, tomatoes, avocados, green beans, etc.) |  | | |  |
|  | 10. Beef, mutton, goat |  | | |  |
|  | 11. Chicken, duck, pigeon |  | | |  |
|  | 12. Liver, heart, kidneys or other organ meats |  | | |  |
|  | 13. Fresh or dried fish or shellfish |  | | |  |
|  | 14. Eggs |  | | |  |
|  | 15. Cheese or other food made from milk (not including yogurt) |  | | |  |
|  | 16. Fat (oil, butter, margarine) |  | | |  |
|  | 17. Chips or other salty snacks |  | | |  |
|  | 18. Biscuits |  | | |  |
|  | 19. Bread or buns |  | | |  |
|  | 20. Candies or chocolates |  | | |  |
|  | 21. Any iron-containing tablet, syrup, or other micronutrient powders or tablets |  | | |  |
|  | 22. Spices/condiments |  | | |  |
|  |  |  | | |  |
|  | How many times did (NAME) eat solid, semi-solid or soft foods other than liquids yesterday, during the day and night?  **Semi-solid** foods such as soft rice, mashed potato, ripe banana, other mashed family foods etc.  **Solid** foods such as rice, bread, potatoes, yams, etc.  Meals include both meals and snacks (other than trivial amounts)  IF DON’T KNOW, ENTER 99. IF REFUSED, ENTER 999. | | Number of times ____________  Don’t know 99  Refused 999 | | |
|  | Of the cooked foods you fed the child yesterday, could you tell me about how many *bowls* (NAME) ate yesterday?  (INTERVIEWER: PLEASE PROBE TO DETERMINE HOW MANY FULL BOWLS PLUS HOW MANY PARTIAL BOWLS (1/4, 1/2, OR 3/4 FULL BOWLS) THE CHILD CONSUMED.) | | Less than ¼ of a small bowl 0  ¼ of a small bowl 1  ½ of a small bowl 2  ¾ of a small bowl 3  One small bowl 4  More than one bowl 5  Other, specify 98  Not given yet 88  Don’t know 99  Refused 999 | | |
|  | ASK THE MOTHER TO BRING THE BOWL SHE USED TO FEED THE CHILD YESTERDAY.  FILL THE BOWL WITH WATER. POUR THE WATER INTO A MEASURING CUP. RECORD THE NUMBER OF MILILITERS (ML) OF WATER. | | _______________ ml of water in the bowl | | |
|  | Would you say that the amount of food (NAME) ate yesterday during the day and night was about the same amount as the child usually eats or a different amount than the child usually eats? | | About the same amount as usual 0>>>211  Different amount than usual 1  Don’t know 99>>211  Refused 999>>211 | | |
| 212a. | Why was the amount of food different? | | Less food than usual because child ill 1  Less food than usual because there was less food in household 2  Less food than usual because fasting 3  Less food than usual because child appetite decreased  4  More food than usual because of celebration 5  More food than usual because more food in  household 6  More food than usual because child had increased appetite 7  Other (specify) 8  Don’t know 99  Refused 999 | | |
|  | Would you say that the type of foods you fed (NAME) yesterday during the day or night were the same or different than usual? | | The same as usual 1  Different than usual, fed more types of foods 2  Different than usual, fed less types of foods 3  Don’t know 99  Refused 999 | | |
|  | At what age did you start giving the following liquids/foods to the child?  NOTE: IF MOTHER FED HER CHILD ANY OF THE FOLLOWING FOOD WITHIN THE FIRST 29 DAYS (LESS THAN 1 MONTHS OF AGE), THIS CAN BE NOTED AS “0” MONTH. | | | | |
|  | **ITEM** | | **RESPONSE** | **MONTH CODES** | |
|  | 1. Water | |  | At “0” month of age 0  At “1” month of age 1  At “2” months of age 2  At “3” months of age 3  At 4 Months of age 4  At 5 Months of ag… 5  At 6 Months of age 6  At 7 Months of age 7  At 8 Months of age 8  At 9 Months of age 9  At 10 Months of age 10  At 11 Months of age 11  At “12” months of age 12  At 24 months of age 24  So on  Not given yet 88  Don’t know 99  Refused 999 | |
|  | 2. Cereals: wheat, millet, sorghum, rice | |  |  |  |
|  | 3. Tubers: yams, cocoyams, potatoes, white sweet potatoes | |  |  |  |
|  | 4. Legume: beans, peas | |  |  |  |
|  | 5. Green leafy vegetables: spinach, ewedu | |  |  |  |
|  | 6. Vegetables such as pumpkin, yellow sweet potatoes, carrots, (vitamin-A rich) | |  |  |  |
|  | 7. Ripe papaya or mango | |  |  |  |
|  | 8. Other fruits and vegetables: Bananas, oranges, apples, avocados, plantains, green beans | |  |  |  |
|  | 9. Meat or poultry: beef, goat, chicken | |  |  |  |
|  | 10. Fish | |  |  |  |
|  | 11. Eggs | |  |  |  |
|  | 12. Peanuts, groundnuts, other nuts | |  |  |  |
|  | 13. Milk (fresh or powdered) and dairy products | |  |  |  |
|  | 14. Infant formula (NAN, SMA Gold, SMA Progress, Peak 123) | |  |  |  |
|  | 15. Packaged infant cereal (Golden Morn, Frisco Cream, Frisco Progress, Quaker Oats) | |  |  |  |
|  | 16. Purchased snack foods (chips, biscuits, chocolate/candies etc.) | |  |  |  |
|  | 17. Purchased drinks (sodas, juices) | |  |  |  |

# MODULE 6: SUPPORT FOR COMPLEMENTARY FEEDING

Now I’m going to ask you some questions about different sources of information about infant and young child feeding and the role (NAME)’s father plays in feeding (NAME).

| What types of help or support has your husband provided related to feeding (NAME)?  **DO NOT READ RESPONSES.**  ***(MULTIPLE RESPONSES POSSIBLE)*** | **INSTRUCTIONS FOR PROGRAMMING:**  **IF PARTICIPANT DESCRIBES NO ROLE, SKIP TO Q622. IF ANY OF THE ROLES LISTED BELOW ARE DESCRIBED, PROGRAM TO RECEIVE ALL ADDITIONAL QUESTIONS RELATED TO ITEMS SELECTED. ALL PARTICIPANTS SHOULD ALSO BE DIRECTED TO 623 AFTER THE OTHER APPROPRIATE QUESTIONS HAVE BEEN ANSWERED.**  He does not provide any help or support related to feeding …0>>622  Provides money for food for the child……………………. 1>>611  Purchases food specifically for the child………… 2>>612 & 613  Gives advice/reminds mother/female relatives about how to feed child ……………………………………………......3>>614 & 615  Monitors how wife/female relatives feeds the child……….4>>616  Feeds the child……………………………………………..5>>617  Teaches the child how to feed him or herself……………...6>>618  Washes the child’s hands before child eats………………...7>>619  Helps me with other chores so that I can feed or prepare food  for the child 8>>620  Other (specify)……………………………………………98>>621  Don’t know………………………………………………99>>622  Refused……………………………………………….... .999>>622 |
| --- | --- |

# MODULE 10: HOUSEHOLD DECISION-MAKING

Now I would like to ask about how you and your partner make decisions

| Who makes the final decision about your husband/partner weekly or monthly income and expenses?  READ RESPONSES 1-4. DO NOT READ “REFUSED.” | Woman 1  Husband/partner 2  Both have the same say 3  Someone else 4  Refuse to answer 999 |
| --- | --- |
| Who makes the final decision about the spending money on large investments, such as buying a cow or plot of land?  READ RESPONSES 1-4. DO NOT READ “REFUSED.” | Woman 1  Husband/partner 2  Both have the same say 3  Someone else 4  Refuse to answer 999 |
| Who makes the final decision about whether you can work outside the home?  READ RESPONSES 1-4. DO NOT READ “REFUSED.” | Woman 1  Husband/partner 2  Both have the same say 3  Someone else 4  Refuse to answer 999 |
| Who makes the final decision about how your husband/partner’s cash earnings are used?  READ RESPONSES 1-4. DO NOT READ “REFUSED.” | Woman 1  Husband/partner 2  Both have the same say 3  Someone else 4  Refuse to answer 999 |
| Who makes the final decision about how your (the woman's) cash earnings are used?  READ RESPONSES 1-4. DO NOT READ “REFUSED.” | Woman 1  Husband/partner 2  Both have the same say 3  Someone else 4  Refuse to answer 999  Not applicable (woman does not have any earnings of her own)…………………………………………………………9999 |
| Who usually makes decisions about what foods to purchase for (NAME): you, your husband/partner, or you and your husband/partner jointly?  READ RESPONSES 1-4. DO NOT READ “REFUSED.” | Woman 1  Husband/partner 2  Both have the same say 3  Someone else 4  Refuse to answer 999 |
| Who usually makes decisions about what to feed (NAME): you, your husband/partner, or you and your husband/partner jointly?  READ RESPONSES 1-4. DO NOT READ “REFUSED.” | Woman 1  Husband/partner 2  Both have the same say 3  Someone else 4  Refuse to answer 999 |

**Data Collection Tools: Fathers’ Questionnaire**

# MODULE 7: SUPPORT FOR COMPLEMENTARY FEEDING

Now I’m going to ask you some questions about your role relating to feeding (NAME).

| **No** | **Questions** | **Code** |
| --- | --- | --- |
|  | What role or roles do you have related to how (NAME) is fed?  DO NOT READ RESPONSES.  ***MULTIPLE RESPONSES POSSIBLE*** | **INSTRUCTIONS FOR PROGRAMMING:**  **IF PARTICIPANT HAS NO ROLE, SKIP TO 713. IF PARTICIPANT DESCRIBES OTHER ROLES WITH LINKS TO OTHER SUBSEQUENT QUESTIONS, PROGRAM THEM TO RECEIVE ALL ADDITIONAL QUESTIONS RELATED TO ITEMS SELECTED. ALL PARTICIPANTS SHOULD ALSO BE DIRECTED TO 713 AFTER THE OTHER APPROPRIATE QUESTIONS HAVE BEEN ANSWERED.**  Have no role 0>>713  Give wife money for food 1>>702  Purchase food for child directly 2>>703 & 704  Give advice/remind wife/female relatives about how to feed  the child 3>>705 & 706  Monitor how wife/female relatives is feeding child 4>>707  Feed the child directly 5>>708  Teach child how to feed him/herself………………..6>>709  Wash child’s hands before eating…………………..7>>710  Help wife/female relatives with other chores so she can feed or prepare food for the child 8>>711  Other (specify)……………………………………98>>712  Don’t know……………………………………….99>>713  Refused………………………………………….999>>713 |

# MODULE 9: HOUSEHOLD DECISION-MAKING

Now I would like to ask about how you and your partner make decisions

|  | Who makes the final decision about your household's weekly or monthly income and expenses?  READ RESPONSES 1-4. DO NOT READ “REFUSED.” | Man 1  Wife 2  Both have the same say 3  Someone else 4  Refuse to answer 999 |
| --- | --- | --- |
|  | Who makes the final decision about the spending money on large investments, such as buying a cow or plot of land?  READ RESPONSES 1-4. DO NOT READ “REFUSED.” | Man 1  Wife 2  Both have the same say 3  Someone else 4  Refuse to answer 999 |
|  | Who makes the final decision about whether your wife can work outside the home?  READ RESPONSES 1-4. DO NOT READ “REFUSED.” | Man 1  Wife 2  Both have the same say 3  Someone else 4  Refuse to answer 999 |
|  | Who makes the final decision about how your wife cash earnings are used?  READ RESPONSES 1-4. DO NOT READ “REFUSED.” | Man 1  Wife 2  Both have the same say 3  Someone else 4  Refuse to answer 999 |
|  | Who makes the final decision about how your (the man's) cash earnings are used?  READ RESPONSES 1-4. DO NOT READ “REFUSED.” | Man 1  Wife 2  Both have the same say 3  Someone else 4  Refuse to answer 999 |
|  | Who usually makes decisions about what foods to purchase for (NAME): you, your wife, or you and your wife jointly?  READ RESPONSES 1-4. DO NOT READ “REFUSED.” | Man 1  Wife 2  Both have the same say 3  Someone else 4  Refuse to answer 999 |
|  | Who usually makes decisions about what to feed (NAME)?  READ RESPONSES 1-4. DO NOT READ “REFUSED.” | Man 1  Wife 2  Both have the same say 3  Someone else 4  Refuse to answer 999 |
